# Noninvasive prenatal testing for chromosome aneuploidies and subchromosomal microdeletions/microduplications in a cohort of 8141 single pregnancies

**DOI:** 10.1186/s40246-019-0198-2

**Published:** 2019-03-12

**Authors:** Hua Hu, Li Wang, Jiayan Wu, Peng Zhou, Jingli Fu, Jiuchen Sun, Weiyi Cai, Hailiang Liu, Ying Yang

**Affiliations:** 1 Second Affiliated Hospital, Army Military Medical University, Chongqing, 400037 China; 2 CapitalBio Technology Inc., Beijing, 101111 China

**Keywords:** Noninvasive prenatal testing (NIPT), Chromosome aneuploidies, Sex chromosome aneuploidy, Subchromosomal microdeletions/microduplications

## Abstract

**Background:**

Noninvasive prenatal testing (NIPT) for fetal aneuploidies by scanning cell-free fetal DNA in maternal plasma is rapidly becoming a first-tier aneuploidy screening test in clinical practices. With the development of whole-genome sequencing technology, small subchromosomal deletions and duplications that could not be detected by conventional karyotyping are now able to be detected with NIPT technology.

**Methods:**

In the present study, we examined 8141 single pregnancies with NIPT to calculate the positive predictive values of each of the chromosome aneuploidies and the subchromosomal microdeletions and microduplications.

**Results:**

We confirmed that the positive predictive values (PPV) for trisomy 13, trisomy 18, trisomy 21, and sex chromosome aneuploidy were 14.28%, 60%, 80%, and 45.83%, respectively. At the same time, we also found 51 (0.63%) positive cases for chromosomal microdeletions or microduplications but only 13 (36.11%) true-positive cases. These results indicate that NIPT for trisomy 21 detection had the highest accuracy, while accuracy was low for chromosomal microdeletion and microduplications.

**Conclusions:**

Therefore, it is very important to improve the specificity, accuracy, and sensitivity of NIPT technology for the detection of subchromosomal microdeletions and microduplications.

## Introduction

In 1997, Lo et al. reported that plasma from pregnant women carrying male fetuses contained cell-free DNA (cf-DNA) derived from the Y-chromosome [1]. Then, cell-free fetal DNA (cff-DNA) was subsequently reported to be used to detect fetal Down’s syndrome and additional fetal aneuploidies in clinical practice [2–4]. Now, noninvasive prenatal testing (NIPT) for fetal aneuploidies by scanning cell-free fetal DNA in maternal plasma is rapidly becoming a first-tier aneuploidy screening test in clinical practice [5, 6]. An increasing number of clinical studies suggest that NIPT has a high sensitivity and specificity for screening trisomies 21 (T21), 18, and 13. Several recent studies have shown that the PPV range of T21 was 65–94%, T18 was 47–85%, and T13 was 12–62% [7–9].

Large or small subchromosomal deletions and duplications are always associated with genetic disorders and syndromes; these are derived from genomic structural changes, such as copy number variants, resulting from abnormal gene dosage with a dramatic influence on gene expression level and phenotype [10]. Currently, the prenatal diagnosis of large subchromosomal deletions and duplications in clinical practice still relies on invasive testing, such as fetal genetic material, through chorionic villus sampling (CVS) and amniocentesis using karyotyping. With the development of whole-genome sequencing technology, smaller pathogenic genomic rearrangements that could not be detected by conventional karyotyping are now able to be detected. Despite the fact that an increasing number of studies have been conducted on the clinical application of NIPT for chromosome aneuploidy detection, knowledge about microdeletion and microduplication syndromes (MMSs) detection has not been given early attention during pregnancy [11]. However, the incidence and severity of the microdeletion and microduplication are higher for Edwards (trisomy 18) and Patau (trisomy 13) syndromes than the 22q11.2 deletion syndrome (known as DiGeorge Syndrome (DGS)), with an incidence of 1 in 992 pregnancies in the low-risk population [12–14]. Therefore, NIPT on subchromosomal microdeletions and microduplications is important for chromosome aneuploidies. This could help identify high-risk pregnancies and offer the possibility of a confirmatory invasive diagnostic test after counseling to offer better clinical management during pregnancy and after birth, where early intervention can potentially improve the quality of life of the newborn.

In the present study, we examined 8152 single pregnancies undergoing NIPT, both for detecting common chromosome aneuploidies, including trisomy 13, 18, and 21 and sex chromosomes, as well as subchromosomal microdeletions/microduplications.

## Materials and methods

### Patients

From March 2016 to May 2017, 8152 pregnant women (Second Affiliated Hospital, Army Military Medical University) opted for NIPT to avoid fetal T13, T18, and T21 aneuploidies. Informed written consent was obtained from all pregnant women who agreed to receive NIPT. Pregnancies with high risks were divided into advanced maternal age, ultrasound abnormalities, poor fertility history, positive serum screening, and other groups.

### Samples preparation and sequencing

Whole blood samples of 5 to 10 mL from pregnant women were collected in EDTA within 8 h or cell-free DNA was collected in BCT tubes (Streck Inc.; Omaha, NE) within 72 h at 4 °C. Afterwards, cfDNA extraction, library construction, quality control, and pooling were performed according to the JingXin Fetal Chromosome Aneuploidy (T21, T18, T13) Testing Kits (CFDA registration permit No. 0153400300). Following the DNA sequencing, 15~20 libraries were pooled and sequenced within ~ 200 bp reads using the JingXin BioelectronSeq 4000 System (CFDA registration permit NO. 20153400309), which is a type of semiconductor sequencer. Sequencing reads were filtered and aligned to the human reference genome (hg19). Fetal DNA concentration was calculated as a quality control using our previously described method [15]. Samples failing the quality criteria of cfDNA extraction, library construction, and sequencing as well as fetal DNA concentration (< 4%) were kicked out.

### Statistics and analysis

Combined GC correction and *Z*-score testing methods were used to identify fetal autosomal aneuploidies, as described previously [16]. Meanwhile, fetal and maternal chromosome copy number variations (CNVs) were classified with our modified Stouffer’s *Z*-score method as described previously [15]. In a previous study, a cutoff value of *Z*-score > 3 was used to determine whether the ratio of the chromosomes was increased and if fetal trisomies 21, 18, and 13 were also present. Here, each chromosome with an absolute value of the *Z*-score greater than 3 was marked with chromosome aneuploidies or microdeletions/microduplications.

### Chromosome karyotype analysis

Chromosome karyotype analysis under sterile conditions was performed on fetal DNA, on cultured amniocytes, and on lymphocytes according to standard protocols. The amniocentesis was performed with the guidance of ultrasound and was centrifuged, inoculated in culture medium, and cultured at 37 °C. Once many circular translucent dividing cells had emerged, colchicine was added and cultured for another 3 h. When the number of circular translucent cells increased, cells were harvested for chromosome preparation. Subsequently, 3 mL of the parents’ peripheral blood was collected with heparin anticoagulation and inoculated in phytohemagglutinin (PHA) culture medium for further karyotype analysis. According to the principle of “An International System for Human Cytogenetic Nomenclature, ISCN2013”, a total of 60 dividing phases were counted using an AI chromosome image analysis system (CytoVision, Switzerland), and 20 karyotypes were analyzed and repeated three times.

### Fish

Fluorescence in situ hybridization (FISH) was used to analyze the interchromosomal rearrangement of the proband. The slides were immersed in Citrisolve for 15 min, jet air-dried, immersed in Lugol solution for 5 min, and immersed in 2.5% sodium thiocyanate for 30 s. The slides were then placed in 10-mM citrate/citric acid solution (pH 6.0) and microwaved on a high setting for 5 min followed by 15 to 45 min in 0.4% pepsin solution (pepsin A/0.9% sodium chloride at pH 1.5) at 37 °C. Ten microliters of FISH reagent (7-μL LSI buffer and 3 μL probe) were placed on each slide, and a cover-slip was added. Slides were then denatured in a Hybrite set at a melting temperature of 80 °C for 5 min and were incubated in a humidified chamber at 37 °C for 12 h. The slides were then washed in 2 × SSC/0.1% NP40 at 70 °C for 2 min and counterstained with 4′,6-diamidino-2-phenylindole dihydrochloride. The cells were analyzed by a microscopist (ML) using a fluorescence microscope equipped with the appropriate filter sets. A minimum of 50 cells and a maximum of 200 cells were scored per case. A minimum of 20 abnormal cells was required for a sample to be considered abnormal [17, 18].

## Results

**Fig. 1:**
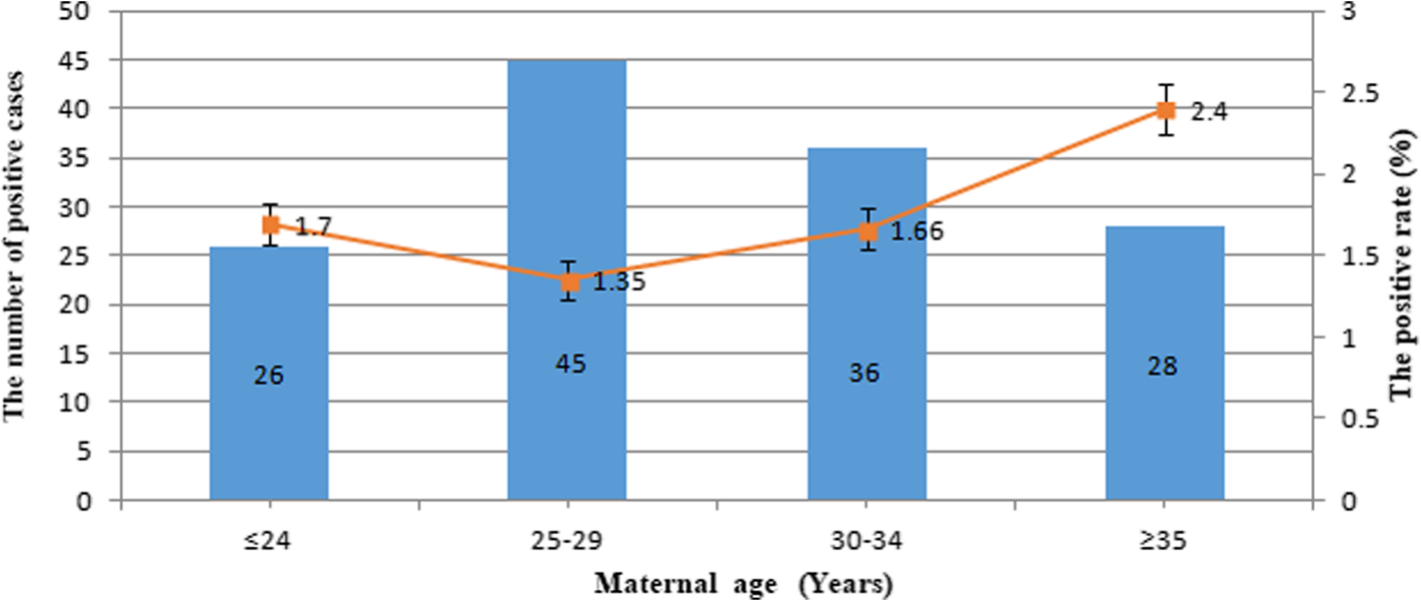
The positive rate of NIPT for aneuploidy and CNV increases with maternal age and the number of positive cases in the four age groups. Binomial test were used to test associations between positive and negative cases in four age groups according to the single pregnancies and the differences were significant (mean_≤ 24_ ± SD_≤ 24_ = 1.9833 + 0.1281, *p* < 0.05; mean_25–29_ ± SD_25–29_ = 1.9867 + 0.11475, *p* < 0.05; mean_30–34_ ± SD_30–34_ = 1.9836 + 0.1271, *p* < 0.05; mean_≥ 35_ ± SD_≥ 35_ = 1.9757 + 0.15413, *p* < 0.05)

Among the 8152 cases undergoing NIPT, we found that due to the low concentration of fetal DNA, 11 cases were not eligible for the next analysis, so the remaining 8141 cases were under-analyzed in the present study. Based on these results, the maternal age for the 8141 pregnancies ranged from 15 to 46 years old. The group aged 25 to 29 years old was the majority (3328, 40.88%) group. Pregnant women older than 35 years were 13.79%. The gestational age at blood sampling ranged from 9 to 34 weeks, and 53.36% of the group had a gestational age from 13 to 16 weeks (see Table 1). The positive rate increased with maternal age and the number of the positive cases in four age groups, as shown in Fig. 1. Binomial test were used to test associations between positive and negative cases in four age groups according to the single pregnancies, and the differences were significant (mean_≤ 24_ ± SD_≤ 24_ = 1.9833 + 0.1281, *p* < 0.05; mean_25–29_ ± SD_25–29_ = 1.9867 + 0.11475, *p* < 0.05; mean_30–34_ ± SD_30–34_ = 1.9836 + 0.1271, *p* < 0.05; mean_≥ 35_ ± SD_≥ 35_ = 1.9757 + 0.15413, *p* < 0.05). Weekly time points of gestation aged from 12 to 26 weeks were tested, but no significant differences were found. In addition to that basic information, we also counted the clinical reasons for NIPT, finding that 13.79% of the group had advanced maternal age more than 35 years (included), and 26 pregnancy cases had ultrasound abnormalities. Other reasons included poor fertility and high risk in serum biochemistry screening.

**Table 1:** Maternal characteristics and gestational age of blood sampling

Maternal age at NIPT (years)	Number	Percent (100%)
≤ 24	1533	18.83
25–29	3328	40.88
30–34	2157	26.50
35–40	1011	12.42
≥ 41	112	1.38
Advanced maternal age (≥ 35 years old)	1123	13.79
Gestational age at NIPT (weeks)		
≤ 8	0	0.00
9–12	916	11.25
13–16	4344	53.36
17–20	1509	18.54
21–24	810	9.95
25–28	347	4.26
≥ 29	212	2.60
Unknown	3	0.04
Range (weeks)	9–34	/

*/* no data

**Fig. 2:**
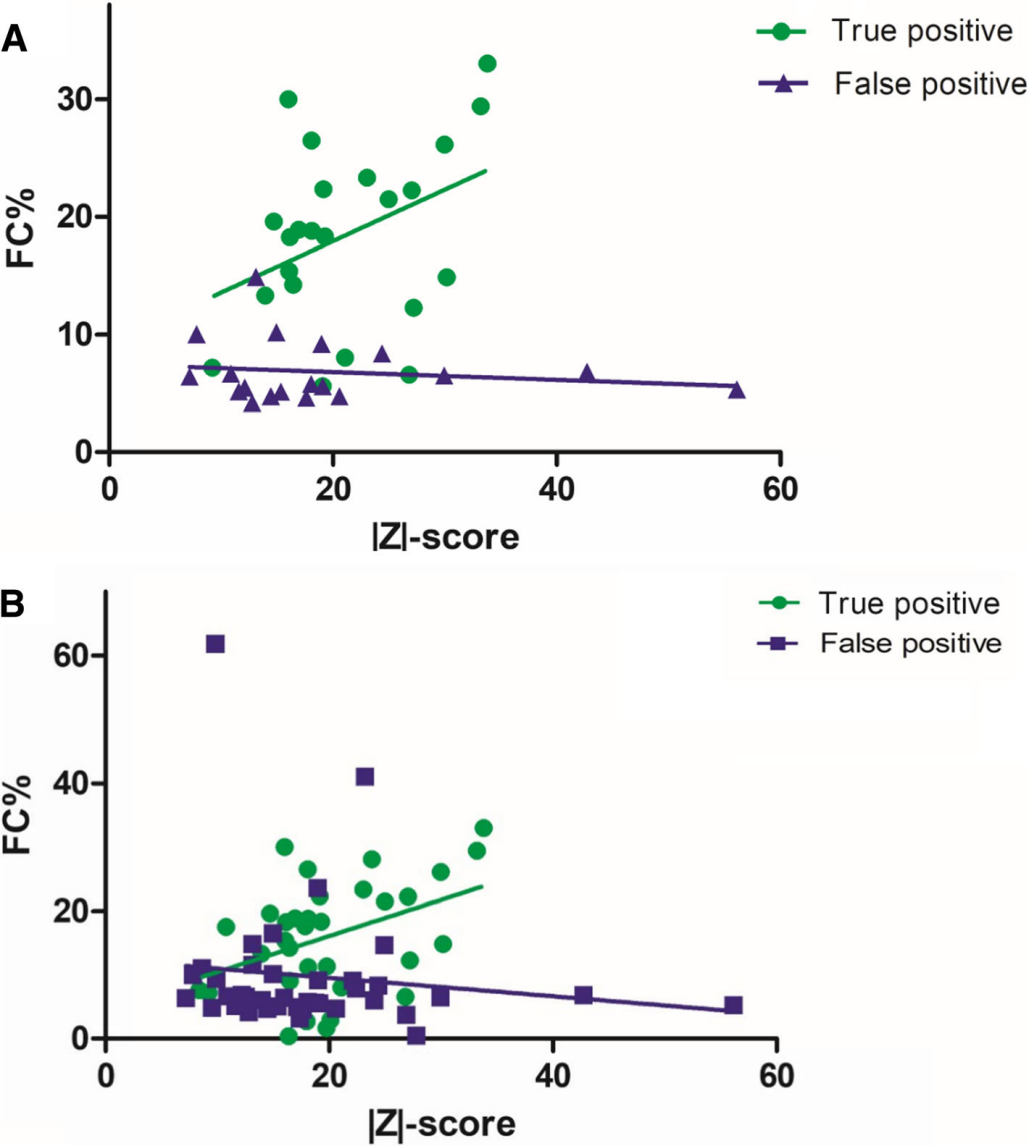
The relationship between fetal DNA concentration and *Z* value in true-positive cases. **a** True positive group of the trisomy sample (*r* = 0.102, df = 21, *p* = 0.076), false positive group of the trisomy sample (*r* = − 0.035, df = 17, *p* = 0.537). **b** True positive group of the total sample (*r* = 0.128, df = 33, *p* = 0.02), false positive group of the total sample (*r* = − 0.003, df = 55, *p* = 0.361)

As shown in Fig. 2, we used linear regression analysis to find the relationship between fetal DNA concentration and *Z* value in the positive cases. We found that there was no obvious linear relationship between them except in true-positive cases with fetal DNA concentration (*r* = 0.128, df = 33, *p* = 0.02). There were 88 (1.08%) positive cases for common chromosome and sex chromosome aneuploidies (SCAs) detection, including 11 cases of trisomy 13, 7 cases of trisomy 18, 36 cases of trisomy 21, and 34 cases of sex chromosome aneuploidy (Table 2). Among them, there were 35 (57.38%) true-positive cases, 26 (42.62%) false-positive cases, and 27 unverified cases that chose to continue gestation or to terminate the pregnancy. For the 35 true-positive cases, there were 20 cases for T21, 1 case for T13, 3 cases for T18, and 11 cases for sex chromosome abnormalities. A total of 26 false-positive cases were normal. Among the 20 cases of T21, two T21 cases were verified as 46,XY,rob(14,21)(q10;q10),+ 21 by amniotic fluid karyotyping analysis.

**Table 2:** NIPT results for chromosome aneuploid and microdeletions/microduplications validated by fetal karyotyping analyses or FISH

No. of chromosome	Aneuploid				Microdeletions and microduplications			
	Positive cases	True-positive cases	False-positive cases	Unverified	Positive cases	True-positive cases	False-positive cases	Unverified
Chr1	/	/	/	/	1	0	0	1
Chr2	/	/	/	/	1	1	0	0
Chr3	/	/	/	/	2	0	1	1
Chr4	/	/	/	/	3	1	1	1
Chr6	/	/	/	/	1	1	0	0
Chr7	/	/	/	/	4	1	2	1
Chr8	/	/	/	/	10	1	8	1
Chr9	/	/	/	/	1	0	1	0
Chr10	/	/	/	/	1	1	0	0
Chr13	11	1 (14.28%)	6 (85.71%)	4	1	/	/	1
Chr14	/	/	/	/	2	1	1	0
Chr15	/	/	/	/	4	1	3	0
Chr16	/	/	/	/	2	0	2	0
Chr18	7	3 (60%)	2 (40%)	2	6	1	0	5
Chr20	/	/	/	/	3	0	2	1
Chr21	36	20 (80%)	5 (20%)	11	3	2	1	0
Chr22	/	/	/	/	2	1	1	0
X or Y	34	11 (45.83%)	13 (54.17%)	10	4	1	0	3
Total	88 (1.08%)	35 (57.38%)	26 (42.62%)	27	51 (0.63%)	13 (36.11%)	23 (63.89%)	15

*/* no data

In addition, we calculated positive cases of chromosomal microdeletions or microduplications as well. Overall, we found 51 (0.63%) cases for chromosomal microdeletions or microduplications, with 13 (36.11%) true-positive cases, 23 (63.89%) false-positive cases, and 15 unverified cases. Of these 23 cases with false positives, the NIPT result of one case showed chromosome 15 microdeletion; however, the amniotic fluid karyotyping analysis prompted 46,XX, 1qh+; the rest were proven normal. Among the 13 cases with true positives, 9 cases occurred because of genetic mutations, while 4 cases were inherited from parents. The karyotypes were confirmed by amniotic fluid karyotyping analysis and FISH (Table 3).

**Table 3:** The true-positive cases of microdeletions/microduplications results

Case	NIPT result CNV location (M) or *Z*-score	CVS	FISH	Pathogenicity
Case 1	2_Z_=17.503	46,XY	47,XN+2[15]/46,XN[85]	/
Case 2	4p16.3-12(dup:0.1Mb-48Mb);8p23.3-23.2(del:1-4Mb)	46,XX,der(8)t(4;8)(p12;p23)pat	/	Wolf-Hirschhorn syndrome [35]
Case 3	6q26-q27(dup:143 Mb-158Mb);6q25.3-27(del:162Mb-171Mb)	46,XN,del(6)(q26)	46,XN,del(6)	Leigh-like syndrome [36]
Case 4	7_Z_=28.110	46,XY	7q31.1 (110.82Mb-111.12Mb)×1	NA
Case 5	8p23.1-11(dup:2Mb-37Mb)	46,XN,der(15)t(8;15)(p11.2;p12)pat	/	Myeloproliferative syndrome [37]
Case 6	10q26(del:127 Mb-133Mb)	46,XY,del(10)(q26.13)	/	Chromosome 10q26 deletion syndrome [38]
Case 7	14q24.3-q32.33(del:44 Mb-105 Mb)	46,XN,del(14)	/	Deafness [39]
Case 8	15q11.2-q13(dup:24 Mb-31Mb)	47,XN,dup(15)(q13)	/	NA
Case 9	18q22.3-q23(del:72 Mb-77.98Mb)	46,XN,del(18)mat	46,XN,del(8)	NA
Case 10	21q11(dup:15 Mb-16Mb)	46,XY	46,XN,dup(21q11.2)(15.4Mb-15.72Mb)×3	NA
Case 11	21q11.2-q21(dup:15 Mb-25Mb)	47, XN,dup(21)(q21.2)mat	/	Usher syndrome [40]
Case 12	22q11(dup:17.46Mb-21.52Mb)	47,XY,der(22)	46,XN,dup(22q11.1-q11.21)(17.42Mb-21.46Mb)×4	DiGeorge syndrome (DGS) [41]
Case 13	X_Z_=-17.652 Y_Z_=-0.782	46,X,del(X)(q21),1qh+	/	/

*/* no data, *NA* no relevant information

## Discussion

Noninvasive prenatal testing of cell-free DNA in maternal plasma, which is a mixture of maternal DNA and a low percentage of fetal DNA, revolutionized the approach to prenatal fetal aneuploidies screening using massively parallel sequencing [19]. A large number of validation studies reporting the sensitivity and specificity of NIPT have been published [3, 20]. Recently, NIPT was also introduced to subchromosomal copy number variations (CNVs), typically less than 5 Mb in size, that could either be inherited from parents with or without symptoms or occur de novo [19, 21]. With the widespread use of whole genome analysis technology, an increasing number of microdeletion and microduplication syndromes connected to certain phenotypes have been diagnosed and researched [22, 23]. In general, microdeletions occurred more frequently than microduplications [7].

In this study, we are the first to use NIPT to screen a large population in the Chongqing area. This NIPT technology uses a semiconductor sequencing platform (SSP) to reliably detect subchromosomal deletions/duplications in women carrying high-risk fetuses. Here, we reviewed the use of NIPT in the context of screening for common chromosome aneuploidies as well as subchromosomal microdeletions and microduplications within a cohort of 8141 single pregnancies (with 11 unqualified samples ruled out) using 4.89 million reads. From our results, we learned that the maternal age for 8141 pregnancies ranged from 15 to 46 years. The group aged 25 to 29 years made up the majority (3328, 40.88%) of the group. Pregnant women over 35 years old were 13.79% of the group. The gestational age at blood sampling ranged from 9 to 34 weeks, and 53.36% of the group had a gestational age from 13 to 16 weeks.

There were a total of 88 (1.08%) positive cases for common chromosome and sex chromosome aneuploidies (SCAs) detected, including 11 cases of trisomy 13, 7 cases of trisomy 18, 36 cases of trisomy 21, and 34 cases of sex chromosome aneuploidies. The positive predictive value (PPV) for common chromosomal aneuploidies in our present study was 57.38%, and for T21, T18, and T13, the PPV was 80%, 60%, and 14.28%, respectively. In several recent studies, the PPV range of T21 was 65–94%, the PPV range of T18 was 47–85%, and the PPV range of T13 was 12–62% [8, 9, 24]. Our results fall within this range. Interestingly, the PPV for SCAs was 45.83%, obviously higher than that of T18 and T13. In this study, the true-positive rate of T13 is relatively low, which may be related to the size of chromosome 13 or the GC ratio on chromosome 13. Zhang et al. showed that such a test was less successful for detecting trisomy 18 and trisomy 13 compared with trisomy 21 [3]. The mixed results may be related to the GC bias caused by sample preparation or sequencing procedures. The differences in the inherent GC content of the chromosomes combined with the sequencer-related GC bias explained the significant correlation between the read coverage and the corresponding GC content. For example, chromosome 13 had a relatively low GC content, and the PCR and sequencing process enriched chromosomes with a higher GC content, leading to a relatively low read coverage for chromosome 13 and thus a negative correlation between read coverage and the GC content among the chromosomes.

Based on a previous study, sex chromosome aneuploidy was frequently suspected from NIPT. The false-positive rate for monosomy X was surprisingly high (91%), and the prediction of other SCAs was more accurate [25]. In Tables 4 and 5, we showed the positive effects of chromosome size, maternal age, and gestational age. We found that the positive results effected from different chromosomes were significantly different, but we could not find statistical significance in chromosome size, maternal age, or gestational age (Table 4). In this study, we found two cases with Robertsonian translocations (der(14;21)). For the most common Robertsonian translocations (der(13;14) and der(14;21)), empirical risk data were summarized by Scriven et al. [26], with a risk of 0.4% for an unbalanced result at a second trimester prenatal diagnosis and an overall risk of miscarriage of approximately 15% in the case of der(13;14). For female carriers of der(14;21), the estimated risk of trisomy 21 at second-trimester prenatal diagnosis is 15%, whereas for male carriers, this risk remains < 0.5%, increasing miscarriage in couples [13, 15]. Cytogenetically, Robertsonian translocation or centric fusion of two long arms of acrocentric chromosomes involving chromosome 21 are the most common structural chromosomal aberrations, which occur with an incidence of ∼ 1 in 1000 in the general population [27]. Although it seems that the prevalence of these structural abnormalities in males and females is similar, a recent study found that women carrying Robertsonian translocations carry the abnormality to the fetus at a rate four times higher than men [27]. Therefore, we recommend that chromosomal karyotypes be detected for parents of such fetuses.

**Table 4:** The positive result effects from maternal age and gestational age

	Trisomy		CNV		Total		*P*
	TP	FP	TP	FP	TP	FP	
Age							
≥ 35	9	4	2	18	11	22	> 0.05^*^
<35	7	8	11	4	18	12	
GA at NIPT (weeks)							
First trimester(9–13 weeks)	0	0	0	0	0	0	/
Second trimester(14–27 weeks)	1	9	13	22	14	31	
Third trimester(≥ 28 weeks)	0	1	0	0	0	1	

*/* no data

^*^Chi-square test in total sample

**Table 5:** The positive result effects from chromosome size

	Positive cases	True-positive cases	False-positive cases	Unverified	*P* ^*^
Trisomy					
Trisomy 13	11	1	6	4	0.05
Trisomy 18	7	3	2	2	
Trisomy 21	36	20	5	11	
Microdeletions and microduplications					
≥ 10 Mb	37	9	19	9	> 0.05
<10 Mb	12	4	3	5	

*Fisher exact probability method

At the same time, we also analyzed the subchromosomal microdeletions and microduplications, finding 51 (0.63%) positive cases but with 13 (36.11%) true-positive cases. As we know, most subchromosomal microdeletions and microduplications occur randomly [23]. In addition, some subchromosomal microdeletions and microduplications had recurrent CNVs, such as 1p36, 3q, 11q23, and 22q11.2 deletion syndromes, with conserved breakpoints that were almost identical even in unrelated individuals [28–31]. In this study, we found 13 (36.11%) true-positive cases for chromosomal microdeletions or microduplications that were validated by chromosome karyotype analysis and FISH. Among the 13 true-positive cases, 9 cases occurred because of genetic mutations, while 4 cases were inherited from parents. By querying the Online Mendelian Inheritance in Man (OMIM) database, 7 cases were identified as syndrome diseases, and 6 cases were pathogenicity unknown (Table 3). The advantages of using diagnostic testing must be balanced with the risk of losing a potential normal pregnancy due to the procedure itself. Broadening the scope of NIPT seems to be the ultimate goal for prenatal screening, thus reducing risks. A major concern is defining for which conditions screening should be offered. The most prevalent microdeletion is 22q11.2, which causes DGS. After Down’s syndrome, DGS is the second most common chromosomal abnormality and cause of congenital heart disease [32]. Although early diagnosis of CNVs can help to avoid years of stress experienced by patients, early treatment can help to improve the symptoms, but CNVs have variable penetrance. In some cases, children with CNVs inherited from parents may have different phenotypes. Therefore, if the NIPT results were positive, validation tests might be considered, such as chromosome karyotype analysis, FISH, or chromosomal microarray analysis.

In this work, we demonstrated the feasibility of performing noninvasive prenatal detection of fetal chromosomal microdeletions and microduplications on a genome-wide level and at 3 Mb resolution. When stratified by CNV size, NIPT identified eight samples with CNVs > 10 Mb and seven samples with CNVs < 10 Mb. Ai-Hua et al. developed a method to identify 71.8% of CNVs using 3.5 million reads, but the performance dropped to 41.2% when CNVs were below 5 Mb [13]. Straver et al. reported the detection of large CNVs (over 20 Mb) with low sequencing depth (0.15–1.66x), which had limited clinical value [33]. Lo et al. reported 64.5% (20/31) accuracy when 4–6 million reads were used to analyze samples with 3 to 42 Mb CNVs [34]. However, if CNVs were smaller than 6 Mb, only 5 in 13 cases were identified. Several studies have claimed benefits; however, we suggest that microdeletions have not demonstrated a sufficiently low false-positive rate to be deemed practical or ethically acceptable, especially considering their low PPV [35]. Because a positive NIPT result should be confirmed using diagnostic techniques and PPV was still lower for some microdeletions, diagnostic testing seems preferable when the goal is to maximize the detection of microdeletion or microduplication syndromes.

NIPT for subchromosomal microdeletions and microduplications was still in its infancy. Until now, no technology had thoroughly validated their tests to a statistically significant level because of the rare occurrence of these chromosomal abnormalities. Therefore, it was very important that the NIPT data must be studied carefully. To be more efficient, NIPT should use increased DNA concentrations or optimized bioinformatics algorithms in CNV to detect CNVs across the whole genome with a very low FPR, as well as high sensitivity and specificity on real-life samples.

## Conclusion

In the present study, we examined 8141 single pregnancies undergoing NIPT. The results of this study indicated that the accuracy of NIPT for T21 detection was higher than that of other chromosome aneuploidies and chromosomal microdeletions/microduplications; it also indicated that the positive predictive value for chromosomal microdeletion/microduplications was still low. Therefore, it was very important to improve the specificity, accuracy, and sensitivity of NIPT technology on the detection of subchromosomal microduplications and microduplications. As noted above, the effect of NIPT may be more pronounced with accurate estimation of the fetal DNA concentration ratio at earlier gestational ages and optimization of the CNV bioinformatics algorithm.

## Abbreviations

cfDNA: Cell-free DNA
cffDNA: Cell-free fetal DNA
CNVs: Copy number variations
CVS: Chorionic villus sampling
DGS: DiGeorge Syndrome
FISH: Fluorescence in situ hybridization
MMSs: Microdeletion and microduplication syndromes
NIPT: Noninvasive prenatal testing
OMIM: Online Mendelian Inheritance in Man
PHA: Phytohemagglutinin
PPV: Positive predictive value
SCA: Sex chromosome aneuploidy
